# MicroRNA-543-3p down-regulates inflammation and inhibits periodontitis through KLF6

**DOI:** 10.1042/BSR20210138

**Published:** 2021-05-21

**Authors:** Wei Li, Junwei Wang, Wenjing Hao, Cuifang Yu

**Affiliations:** 1 Department of Stomatology, The Affiliated Hospital of Qingdao University, No. 16 Jiangsu Road, Shinan District, Qingdao 266000, Shandong, China; 2 Department of Stomatology, Qingdao Women and Children’s Hospital. No. 6 Tongfu Road, Shibei District, Qingdao 266000, Shandong, China; 3 College of Food Science and Engineering, Qingdao Agricultural University. No. 700 Changcheng Road, Chengyang District, Qingdao 266109, Shandong, China

**Keywords:** Kruppel-like factor 6, LPS: lipopolysaccharide, normal control

## Abstract

MicroRNA-543-3p (miR-543-3p) has been reported to be involved in many human disease’s progression, but its role in inflammation is still unclear. After bacterial infection, innate immune cells are activated to trigger inflammation by recognizing lipopolysaccharide (LPS) on the bacterial outer membrane. In our research, it showed that miR-543-3p was down-regulated in LPS-treated periodontal ligament cells (PDLCs). And it mediated the apoptosis of PDLC induced by LPS, which may be involved in periodontitis development. Besides, up-regulation of miR-543-3p alleviated the inflammatory damage induced by LPS. Furthermore, our research demonstrated Kruppel-like factor 6 (KLF6) served as a direct downstream target of miR-543-3p to play a vital role in periodontitis. Simply put, these findings suggest that miR-543-3p could down-regulate inflammation and inhibit periodontitis by targeting KLF6, and it provides a new insight into the molecular mechanism of periodontitis, which may be helpful for the early diagnosis and treatment of this disease.

## Introduction

Periodontitis, a complex infectious disease with multiple etiologies and causes [1], is defined as the pathological loss of periodontal ligaments and alveolar bone [2]. It is reported that under the stimulation of lipopolysaccharide (LPS) of periodontal pathogens (including *Porphyromonas gingivalis*), differential gene expression appears in the neutrophils of healthy people [3,4]. The concepts of induction, regulation and effector functions of periodontal immune/inflammatory response (including cytokines) have been reported [5]. The inflammatory factors (such as interferon γ (IFN-γ), interleukin (IL) 17 (IL-17), and tumor necrosis factor α (TNF-α)) activate the immune defense pathogens of M1 macrophages, but pro-inflammatory cytokines released for a long time or overproduction can activate osteoclasts (monocytes/macrophages cell line) and matrix metalloproteinase (collagenase). Anti-inflammatory factors (such as IL-10, IL-13, and IL-4) facilitate the activation of M2 macrophage cells and participate in the maturation, proliferation, and isotype conversion of B cells, thereby involving in antibody production and dental control of periodontitis [6].

In epigenetics, miRNAs modulate the expression of genes in cells [7]. The six to eight nucleotides at the 5′ end of the miRNA sequence specifically bind to the non-coding region at the 3′ end of the target gene mRNA, thereby regulating the transcription expression of gene mRNA or inhibition of mRNA degradation [8,9]. It has been reported that miR-543 acts as a promoter of osteogenesis in human periodontal ligament-derived stem cell (hPDLSCs) by suppressing its target gene *TOB2* [10]. Moreover, miR-543-5p inhibits NF-κB pathway and reduces the release of inflammatory factors, and ameliorates nerve regeneration, and ultimately promotes hindlimb locomotor function [11]. In addition, miR-543-3p leads to the improvement of neuron protection and locomotor function via attenuating inflammatory reaction and cell apoptosis [12]. MiR-543-3p enhances anti-inflammatory and myeloid-derived suppressor cell (MDSC) regulatory genes, such as IL-10, CCL11, and its receptors CCR5, and CXCR2 [13]. However, the mechanism of miR-543-3p for the specific development of periodontitis remained unclear.

Kruppel-like factor 6 (KLF6), a type of zinc finger transcription factor in the Kruppel-like factor family, is involved in regulating cell apoptosis and other physiological process. In prostate cancer, liver cancer and other cancers, the expression of KLF6 is significantly decreased, and KLF6 overexpression inhibits the progression of the above-mentioned tumors, and promotes tumor cell apoptosis [14,15]. KLF6 regulates the expression level of downstream gene *p21* through a p53-independent signal pathway, and the latter promotes cell apoptosis [16]. When KLF6 is abnormally expressed in tumors, it leads to down-regulation of the downstream gene *p21* expression, which hinders the apoptotic process of tumor cells and is in a state of malignant proliferation, leading to continuous development of tumor cells [14].

At present, the mechanism of microRNAs that played a regulatory role in the inflammatory response had not been reported. In this article, we explored the specific role of miR-543-3p on the inflammatory response and found that KLF6 was a direct target of miR-543-3p. The function of miR-543-3p/KLF6 was determined to patriciate in the occurrence of inflammation in the periodontal ligament cells (PDLCs) induced by LPS, providing a new insight for the clinical treatment of periodontitis.

## Materials and methods

### Cell culture and transfection

PDLCs were purchased from the American Type Culture Collection (Manassas, VA, U.S.A.) and cultured in DMEM/F12 medium containing 10% fetal bovine serum and 1% antibiotics (100 μg/ml streptomycin and 100 U/ml penicillin) at 37°C with 5% CO_2_.

For cell transfection, a serum-free medium was used to dilute the transfection reagent Lipofectamine® 3000 (Invitrogen, CA, U.S.A.). The normal control shRNA (NC) and KLF6 targeting shRNA were diluted at 50 μmol/l using a serum-free medium. shRNA was then mixed with the transfection reagent. After 12-h transfection, the status of transfected cells was observed, and when the cell status was confirmed normal, serum-free medium was replaced with complete medium. After continuous culture for 48 h, RNA was isolated from the cells, and the transfection efficiency was detected by quantitative reverse transcription polymerase chain reaction (qRT-PCR). At the same time, two plasmids, the normal control (NC) plasmid and the overexpression plasmid KLF6, designed and constructed by GenePharma (Suzhou, China), were transfected into LPS-treated cells.

### Mass concentration test

Cells (1 × 10^6^/ml) were inoculated in 96-well culture plates. According to the experimental groupings, different concentrations of LPS (Sigma, CA, U.S.A.) were added to each well of the low-, medium-, and high-dose LPS groups to make the final concentration 0.1, 1, and 5 mg/l; the control group was added with the same amount of medium and incubated at 37°C in 5% CO_2_ for 24 h. The supernatant was aspirated and the contents of TNF-α, IL-1β in the supernatant were detected according to the ELISA kit instructions (Shanghai, China).

### qRT-PCR 

TRIzol was used to isolate RNA from cells or tissues. After the RNA was completely dissolved, the 5× PrimeScript® RT Master Mix kit was used for reverse transcription (10 μl). qRT-PCR was carried out by SYBR Premix ExTaq™ (Takara, Otsu, Japan) on StepOnePlus™ Real-Time PCR System (Applied Biosystems, Thermo Fisher Scientific). The primers used for real-time qPCR were as follows: miR-543-3p: 5′-CGGGGGTAATTTTATGTATAAGCTAGT-3′; KLF6: forward 5′-TGAGCCTGGTGAGCCCG-3′, reverse 5′-TCTCGCCAGGTCTTCCAGG-3′; GAPDH: forward 5′-TATGATGATATCAAGAGGGTAGT-3′, reverse 5′-TGTATCCAAACTCATTGTCATAC-3′; U6: forward 5′-GCTTCGGCAGCACATATACTAAAAT-3′, reverse 5′-CGCTTCACGAATTTGCGTGTCAT-3′. The 2^−ΔΔ*C*_t_^ method was used to assess and normalize relative each gene expression levels.

### Cell counting kit 8 assay

Cell Counting Kit 8 (CCK-8; Apexbio, HOU, U.S.A.) was used to detect PDLC growth. Briefly, the cells were incubated in 96-well-plate for 24, 48, or 72 h. Then, the cells were treated with the CCK-8 solution. Next, the plate was incubated at 37°C for 3 h. The optical density (OD) value was detected at 450 nm by ultraviolet spectrophotometer (Thermo Fisher Scientific, Inc.).

### EdU assay

The cells (8 × 10^3^/100 μl) were inoculated into 96-well plate. When the cells adhered to the wall, 100 μl of 50 μM EdU liquid medium was added to each well, and the culture plate was placed in incubator at 37°C for 2 h. After fixing, EdU at 1:1000 in serum-free DMEM/F12 medium was added, and the cells were incubated for 2 h. Steps were guided by the directions of EdU kit (YIHX Biotechnology, Beijing, China). Three random fields were observed and imaged with an inverted fluorescence microscope.

### Flow cytometry

Cells in both groups were washed with PBS, resuspended by appending with 100 μl Propidium Iodide (PI) orderly, and then mixed well to avoid light and allowed to stand for 15 min. Annexin V-fluorescein isothiocyanate/propidium iodide apoptosis assay kit (Abcam, Cambridge, MA, U.S.A.) was used to detect apoptotic cells. As previously, FACScan flow cytometry system (Becton Dickinson, CA, U.S.A.) was used for apoptosis analysis.

### Dual-luciferase reporter gene assay

Two target fragments including wildtype (WT) and miR-543-3p binding site mutant (MUT) were constructed and they were inserted into pGL3 vector, and the report vectors of KLF6-WT and KLF6-Mut were constructed as before [1]. The WT 3′ untranslated region (UTR) and mutants of KLF6 were constructed through the mutation of the miR-543-3p binding site in KLF6 3′UTR, and the downstream luciferase gene of psiCHECK-2 luciferase vector was cloned (Promega, Madison, WI, U.S.A.). After the transfection plasmid was added to cells using Lipofectamine 3000 (Invitrogen) for 48 h, the Dual-Luciferase Reporter Assay System was used to detect *Renilla* luciferase in cell lysate (Promega, Madison, WI, U.S.A.).

### Western blot analysis

Bicinchoninic acid (BCA) kit (Wuhan Bost Biotechnology Company, Hubei, China) was used to extract the total proteins required for the experiment and detect its concentration. Then 30 mg/well of loading buffer was added to the extracted proteins and boiled in a 95°C water bath for 5 min. After that, it was first separated by 10% polyacrylamide gel electrophoresis (Wuhan Bode Biotechnology, Hubei, China), and proteins were transferred to polyvinylidene fluoride (PVDF) transmembrane. Finally, the transferred transmembrane was placed in skim milk for 1 h. These bands were incubated with primary mouse antibodies PCNA (PC10), Bax (2D2), Bcl-2 (C-2), Ki-67 (Ki-67), β-actin (C-2), NF-κB (E-10) (1:1000, Santa Cruz, CA, U.S.A.), TNF-α (EPR19147) (1:1000, Abcam, MA, U.S.A.), and rabbit antibodies cleaved caspase-3 (Asp^175^), cleaved caspase-9 (Asp^315^) (1:1000, Cell Signaling, MA, U.S.A.) at 4°C overnight. Then, these bands were incubated with the corresponding horseradish peroxidase (HRP)-conjugated secondary anti-rabbit or anti-mouse IgG (1:2000, Santa Cruz, CA, U.S.A.) for 1–2 h. The dual-color infrared fluorescence scanning imaging system (Odyssey, LI-COR, NE, U.S.A.) was used to capture images, and then ImageJ 1.52v imaging analysis software (NIH, Bethesda, MD, U.S.A.) was used for analysis.

### Statistical analysis

Results were collected from three independent experiments. All the results were presented as means ± SD. Results were analyzed with one-way ANOVA and *t* test using GraphPad Prism 7.0 (GraphPad Inc., San Diego, CA, U.S.A.). *P*<0.05 was considered statistically significant.

## Results

### MiR-543-3p is down-regulated in LPS-treated PDLCs

**Figure 1:**
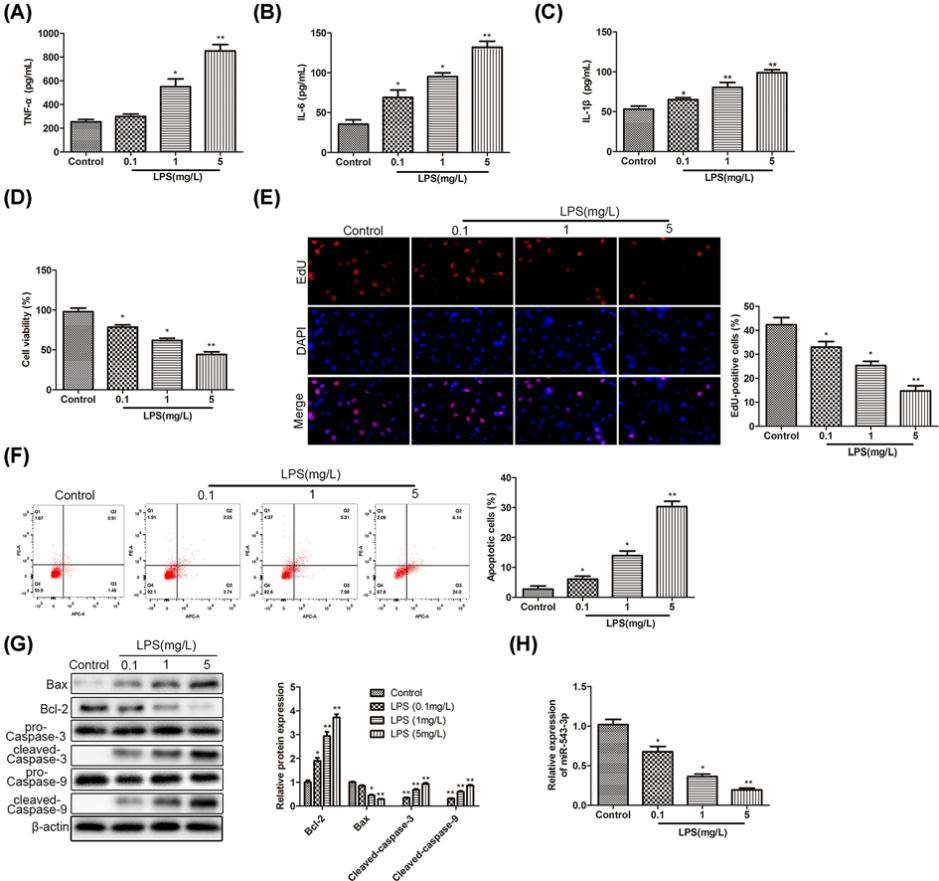
MiR-543-3p is down-regulated in LPS-treated PDLCs (**A–C**) Different concentrations (0.1, 1, and 5 mg/l) of LPS was used to treat cells, and the levels of inflammatory cytokines were detected. Results are presented as the means ± SD from three independent experiments. Significance was determined using Student’s *t* test. *P<0.05, **P<0.01. (A) TNF-α. (B) IL-1β. (C) IL-6. (**D**,**E**) The cell viability in cell models treated with LPS (0.1, 1, and 5 mg/l) was detected by CCK-8 assay and EdU assay, and the bar graph showed percentage of cell viability and EdU-positive cells in control group *vs.* LPS-treated group. Data correspond to the means ± SD of at least three independent experiments, *P<0.05, **P<0.01. (**F**) Flow cytometry was carried out to detect the apoptosis level of cells treated with LPS (0.1, 1, and 5 mg/l). Data represent percentage of cell apoptosis (means ± SD) of three independent experiments. *P<0.05, **P<0.01. (**G**) Western blot analysis showed the proteins level of Bax, Bcl-2, cleaved caspase-3, and cleaved caspase-9 in the cells treated with LPS (0.1, 1, and 5 mg/l). Protein bands were quantified by ImageJ software, which was normalized to β-actin levels. The data were presented with the means ± SD of three independent experiments. Significance was determined using Student’s *t* test. *P<0.05, **P<0.01. (**H**) The expression level of miR-543-3p expression in control group *vs.* LPS-treated group with different concentrations (0.1, 1, and 5 mg/l). The quantitative results were the means ± SD of three independent experiments. *P<0.05, **P<0.01.

To explore the effects of LPS, different concentrations of LPS (0.1, 1, and 5 mg/l) were used to stimulate the cells, and the mass concentrations of TNF-α, IL-6, and IL-1β were detected 24 h later. As shown in Figure 1A–C, the results illustrated that with the increase in LPS concentration, the concentrations of inflammatory cytokines were increased significantly. However, CCK-8 assay demonstrated that the cell viability was inhibited by LPS in a concentration-dependent manner (Figure 1D). Besides, we detected the cell proliferation with EdU staining and found that the percentage of EdU-positive cells was decreased by LPS treatment (Figure 1E). Next, flow cytometry suggested that cell apoptosis was promoted with the increase in LPS concentration in PDLCs (Figure 1F). Furthermore, we detected the expression of apoptosis-associated proteins in LPS-treated cells by Western blot analysis. We found that the expression of Bcl-2, cleaved caspase-3, and cleaved caspase-9 was up-regulated with the increase in LPS concentration compared with the control group (Figure 1G), while the expression of Bax was decreased in turn. Interestingly, in the LPS-induced periodontitis model, the miR-543-3p, reported function in inflammatory reaction, was significantly decreased during LPS-induced periodontitis (Figure 1H). The above results revealed that miR-543-3p was down-regulated in LPS-treated PDLCs and it suggested that miR-543-3P may be involved in periodontitis development.

### Up-regulation of miR-543-3p alleviates the inflammatory damage induced by LPS

**Figure 2:**
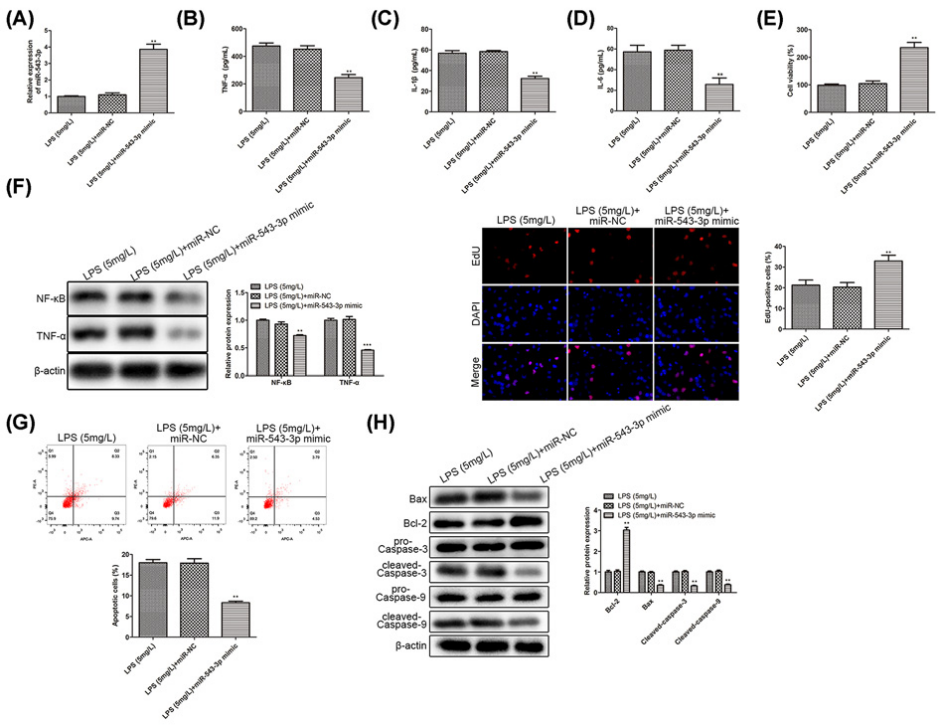
Up-regulation of miR-543-3p alleviates the inflammatory damage induced by LPS All data correspond to the means ± SD of three independent experiments. Statistical significance was calculated by the Student’s *t* test. (**A**) qRT-PCR was used to detect the overexpression efficiency of miR-543-3p in cells treated with 0.1 mg/l LPS, LPS (0.1 mg/l) + miR-NC mimics and LPS (0.1 mg/l) + miR-543-3p. **P<0.01. (**B–D**) The concentrations of TNF-α, IL-1β, IL-6 in cells treated with 0.1 mg/l LPS, LPS (0.1 mg/l) + miR-NC mimics and LPS (0.1 mg/l) + miR-543-3p mimics, **P<0.01. (**E**) The cell viability of cells treated with 0.1 mg/l LPS after overexpression of miR-543-3p was detected by CCK-8 assay, and the bar graph showed percentage of cell viability in LPS group, LPS + miR-NC mimics group and LPS + miR-543-3p mimics group, *P<0.05. (**F**) PDLCs were treated with LPS (0.1 mg/l), LPS (0.1 mg/l) + miR-NC mimics and LPS (0.1 mg/l) + miR-543-3p mimics. Then cells were harvested at 48 h. Whole cell extracts were subjected to immunoblot analysis with NF-kB and TNF-α. β-actin was used as loading control (**P<0.05; ***P<0.01). In addition, the cell viability of cells treated with LPS after overexpression of miR-543-3p was detected by EdU assay, and the bar graph showed percentage of EdU-positive cells in LPS group (0.1 mg/l), LPS (0.1 mg/l) + miR-NC mimics group and LPS (0.1 mg/l) + miR-543-3p mimics group, **P<0.01. (**G**) Apoptosis of cells treated with 0.1 mg/l LPS in each group was detected by flow cytometry. The bar graph showed the percentage of apoptotic cells in each group, **P<0.01. (**H**) Western blot analysis showed the proteins level of Bax, Bcl-2, cleaved caspase-3, and cleaved caspase-9 in LPS (0.1 mg/l)-treated group, LPS (0.1 mg/l) + miR-NC mimics group and LPS (0.1 mg/l) + miR-543-3p mimics group, **P<0.01.

To probe the roles of miR-543-3p on the proliferation and apoptosis in PDLCs, miR-543-3p mimic and miR-NC were used to treated LPS-induced periodontitis and transfection efficiency was shown in Figure 2A. Similarly, the mass concentrations of TNF-α, IL-1β, and IL-6 were detected and significant decreases were observed in the miR-543-3p mimic-treated group (Figure 2B–D). Also, it showed that the level of NF-κB expression was significantly decreased in the group of LPS combined with miR-543-3p mimics in comparison with control groups (Figure 2F). These results suggested that up-regulation of miR-543-3p alleviates the inflammatory damage induced by LPS. CCK-8 assay and EdU staining showed that the cell viability was activated and the EdU-positive cells were increased in miR-543-3p mimics group (Figure 2E,F), suggesting that miR-543-3p mimics retarded LPS-inhibited cell proliferation. Next, flow cytometry demonstrated that overexpression of miR-543-3p prevented LPS-induced cell apoptosis (Figure 2G). Furthermore, Western blot results showed that compared with the control group (only add LPS), overexpression of miR-543-3p increased the expression of Bcl-2 (Figure 2H), while decreased the expression of Bax, cleaved caspase-3, and cleaved caspase-9. All these results confirmed that overexpression of miR-543-3p activated the proliferation and prevented apoptosis of LPS-treated PDLC.

### KLF6 serves as a direct downstream target of miR-543-3p

**Figure 3:**
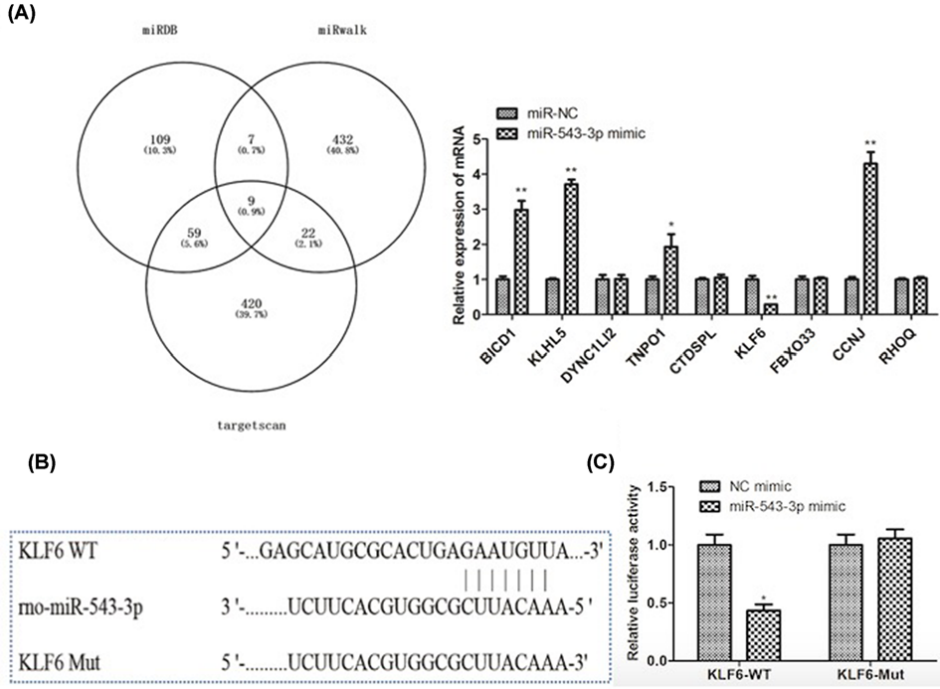
KLF6 serves as a direct downstream target of miR-543-3p (**A**) The possible downstream targets of miR-543-3p (ENCORI, TargetScan, and miRWalk) by three kinds of silica gel prediction algorithms. The mRNA expression level of relative target genes. All data were means ± SD of at least three independent experiments. Statistical significance was calculated by the Student’s *t* test. *P<0.05, **P<0.01. (**B**) The binding sites of miR-543-3p and KLF6 were predicted by a bioinformatics website. (**C**) The targeting relationship between miR-543-3p and KLF6 was determined by dual luciferase report assay. Results are presented as the means ± SD from three independent experiments. Statistical significance was calculated by the Student’s *t* test: *P<0.05.

To explore the mechanism through which miR-543-3p activated the cell proliferation and apoptosis, we further predicted the possible downstream targets of miR-543-3p by ENCORI, TargetScan and miRWalk, and several target genes were predicted. In order to find out the target gene downstream miR-543-3p, we performed qRT-PCR analysis and found that miR-543-3p mimics specifically down-regulated the level of KLF6 (Figure 3A), then, we predicted the potential miR-543-3p binding site in KLF6 (Figure 3B). To verify their connection, the luciferase reporter vectors 3′UTR of KLF6-WT or KLF6-MUT were constructed and was transfected into cells. MiR-543-3p mimic greatly reduced the luciferase activities of KLF6-WT but had no effect on KLF6-MUT (Figure 3C). It was verified that KLF6 was a direct target of miR-543-3p.

### MiR-543-3p/KLF6 axis mediates in the development of inflammation in the LPS-treated PDLCs

**Figure 4:**
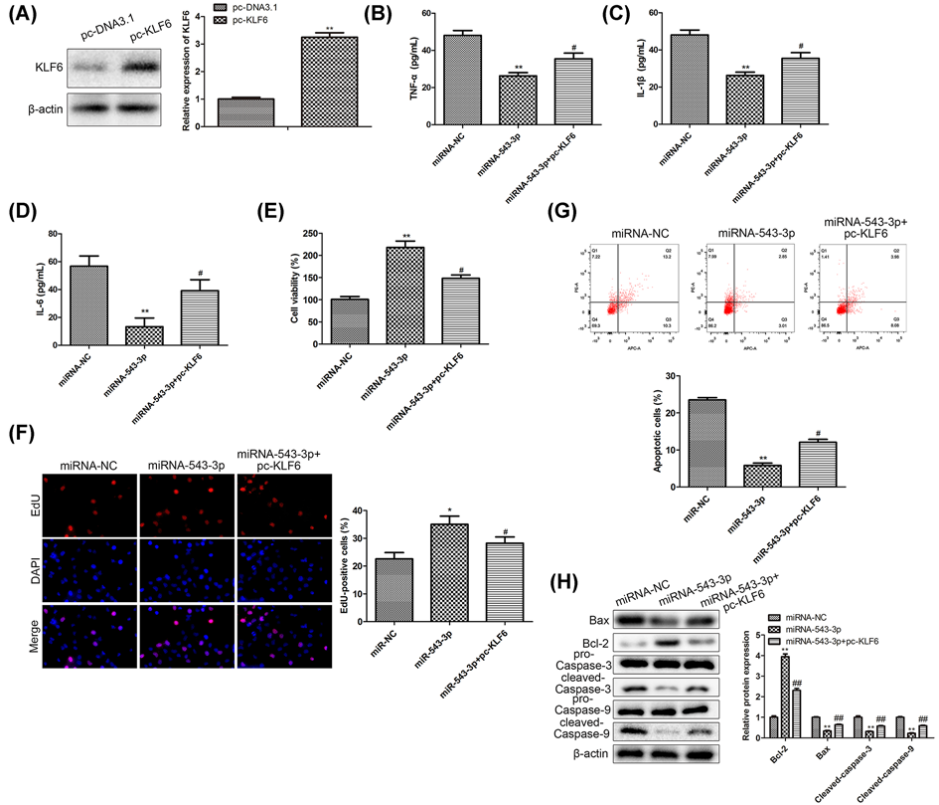
MiR-543-3p/KLF6 axis mediates in the development of inflammation in the LPS-treated PDLCs The results represent summary data from three independent experiments (means ± SD). Statistical significance was calculated by the Student’s *t* test at a probability level. (**A**) qRT-PCR was used to detect the expression of miR-543-3p and KLF6 in LPS-treated cells, **P<0.01. (**B–D**) The concentrations of TNF-α, IL-1β, IL-6, **P<0.01, *^#^P*<0.05. (**E**) The cell viability of LPS-treated cells after knocking down LPS was detected by CCK-8 assay. The diagram showed changes in each group, **P<0.01, *^#^P*<0.05. (**F**) EdU assay was used to instruct proliferation ability of LPS-treated cells. The bar graph showed percentage of EdU-positive cells in miR-NC group, miR-543-3p mimic group and miR-543-3p mimic +pc-KLF6 group, *P<0.05, *^#^P*<0.05. (**G**) Apoptosis of in each group was detected by flow cytometry. The bar graph showed the percentage of apoptotic cells in each group, **P<0.01, *^#^P*<0.05. (**H**) Western blot analysis showed the proteins level of Bax, Bcl-2, cleaved caspase-3 and cleaved caspase-9 in miR-NC group, miR-543-3p mimic group and miR-543-3p mimic+ pc-KLF6 group, **P<0.01, *^##^P*<0.01.

To detect whether KLF6 indeed mediates the effects of miR-543-3p on PDLCs, we overexpressed the KLF6 by using pc-KLF6. The overexpression efficiency was detected by qRT-PCR and was presented in Figure 4A. The mass concentrations of TNF-α, IL-1β, and IL-6 were reduced by miR-543-3p transfection in PDLCs, whereas KLF6 overexpression rescued the levels of these pro-inflammatory cytokines down-regulated by miR-543-3p mimics (Figure 4B–D). CCK-8 assay showed the KLF6 overexpression could block the pro-proliferation role of miR-543-3p, meanwhile EdU staining also indicated the same conclusion, as KLF6 overexpression decreased the EdU-positive PDLCs induced by miR-543-3p mimic (Figure 4E,F); these results suggested that KLF6 inhibited cell proliferation induced by miR-543-3p mimic. In addition, flow cytometry indicated that the apoptosis of the LPS-treated PDLCs was reduced in miR-543-3p mimic group, whereas KLF6 reversed this anti-apoptosis induced by miR-543-3p mimic (Figure 4G). Furthermore, we detected the levels of apoptosis-associated proteins by Western blot and the expression of Bcl-2 was increased by miR-543-3p-overexpression compared with control group, consistently the levels of apoptosis markers-Bax, cleaved caspase-3, and cleaved caspase-9 were down-regulated, and KLF6 partially blocked the anti-apoptosis effect of miR-543-3p. (Figure 4H). Taken together, miR-543-3p play a critical role in the progression of periodontitis by modulating the expression of KLF6. Also, the present findings could provide evidence that KLF6 indeed mediate the effects of miR-543-3p on pro-inflammatory cytokines, cell viability and apoptosis of PDLCs. These findings should be followed by a detailed investigation utilizing knockdown of KLF6 in PDLCs and control cells with/without LPS treatment to identify the function of KLF6 in the development of inflammation in the LPS-treated PDLCs in many aspects.

## Discussion

Periodontitis is a complex infectious disease with multiple etiologies and causes [1]. With the changes in eating habits, the incidence of periodontitis has gradually increased which seriously threatens the health and quality of life of residents. It is necessary to study the occurrence and development mechanism of periodontitis in order to find an effective treatment. We performed the present study to investigate which role the miR-543-3p/KLF6 axis plays in periodontitis, and found that KLF6 functions in the progression of periodontitis by modulating miR-543-3p.

In some cancer cells and neurons, miR-543-3p plays a very vital regulatory role, but the function of miR-543-3p on periodontitis has not been reported. miR-543-3p plays an important role in promoting the proliferation and stem cell-like phenotype of bladder cancer [17]. Others have reported that knockdown of miR-543-3p rescued the function of glutamate transporter type 1 (GLT-1) in the Parkinson's disease (PD) model and alleviate dyskinesia, suggesting that knockdown of miR-543-3p may be used as a potential therapeutic target for PD [18]. There are few studies on miR-543-3p in the inflammatory response, and no abnormal expression of miR-543-3p in periodontitis has been found. It has been reported that miR-543 acts as a promoter of osteogenesis in hPDLSCs by targeting TOB2 [10]. MiR-543-5p inhibits NF-κB pathway, reduces the inflammatory factors, ameliorates nerve regeneration, and ultimately promotes hindlimbs locomotor function [11]. In our study, miR-543-3p was down-regulated in LPS-treated PDLC, and overexpression of miR-543-3p down-regulates LPS-treated inflammatory response.

In a variety of human cells, KLF6 plays a very critical role in the inflammation [19,20]. KLF6 is located in the nucleus and is a nuclear transcription factor, which takes part in the growth, differentiation, and proliferation of cells. Actually, the KLF6 also promotes cell apoptosis and regulates cell senescence and other life activities. KLF6 often exerts a tumor suppressor effect in cells, and its abnormal expression leads to abnormal cell growth and malignant proliferation. Studies have shown that in oral cancer tissues, the expression of KLF6 is significantly reduced. When it is overexpressed in oral cancer cell lines, the cell proliferation ability is significantly reduced, the apoptosis rate is significantly increased, and the cell migration ability is also significantly reduced [21]. KLF6 participates in cell growth regulation by regulating the expression of the downstream gene *p21* [22]. Recent studies have found that KLF6 regulates the activity of the p21WAF1/CIP1 signal pathway through the p53-independent signal pathway to inhibit cell growth and proliferation [23]. In addition, KLF6 regulates the cell cycle process through the cyclin-dependent signal pathway [24]. Some current studies have shown that the expression of KLF6 in a variety of cells can be regulated by the expression of different miRNAs, and may be involved in the occurrence and development of a variety of tumors [25,26]. Our research revealed that miR-543-3p directly targeted the 3′-UTR of KLF6 mRNA and decreased its expression. And KLF6 overexpression attenuated the anti-inflammatory effect of miR-543-3p in periodontitis.

In summary, our research demonstrated that miR-543-3p participates in the occurrence and progression of periodontitis by targeting KLF6. At present, the molecular mechanism that miR-543-3p regulates the expression of KLF6 and affects the occurrence and development of periodontitis. Considering several target genes of miR-543-3p, it will be further investigated in due course whether they also play a significant role in the anti-inflammatory role of miR-543-3p. The present study provided a new direction for the study of the molecular mechanism of the periodontitis progression, as well as the research of the disease. The new theoretical basis might help the early diagnosis of periodontitis and provide new entry points and research ideas for the treatment of this disease.

## Data Availability

All data generated or analyzed during the present study are included in this published article.

## Abbreviations

CCK-8: cell counting kit 8
EdU: 5-Ethynyl-2′-deoxyuridine
GLT-1: glutamate transporter type 1
hPDLSC: human periodontal ligament-derived stem cell
IL: interleukin
KLF6: Kruppel-like factor 6
LPS: lipopolysaccharide
MDSC: myeloid-derived suppressor cell
NC: normal control
PD: Parkinson's disease
PDLC: periodontal ligament cell
qRT-PCR: quantitative reverse transcription polymerase chain reaction
TNF-α: tumor necrosis factor α
UTR: untranslated region
WT: wildtype
